# The tryptophan prenyltransferase ComQ from *Bacillus subtilis* 168 can prenylate daptomycin at Trp1

**DOI:** 10.1111/febs.70578

**Published:** 2026-05-02

**Authors:** Yanli Xu, Maximilian J. L. J. Fürst, Oscar P. Kuipers

**Affiliations:** ^1^ Department of Molecular Genetics Groningen Biomolecular Sciences and Biotechnology Institute, University of Groningen The Netherlands; ^2^ Molecular Enzymology, Groningen Biomolecular Sciences and Biotechnology Institute, University of Groningen The Netherlands

**Keywords:** *Bacillus subtilis* 168, ComQ_168_, daptomycin, farnesylation, modeling

## Abstract

Prenylation is a common natural modification that enhances peptide bioactivity. Identifying prenyltransferases with broad substrate specificity is vital for enabling the modification of diverse compounds. In *Bacillus subtilis* 168, the tryptophan at Position 3 from the C terminus of the ComX_168_ pheromone is farnesylated by ComQ_168_, producing an inducer of natural competence. While ComQ_168_'s native substrate, ComX, is a linear peptide, many bioactive cyclic peptides like daptomycin and polymyxin also contain long lipid tails, similar to farnesylated ComX. This study investigates whether ComQ_168_ can be used as a synthetic biology tool to farnesylate cyclic antimicrobial peptides. ComQ_168_ was cloned from *B. subtilis* 168, expressed in *E. coli*, and purified in soluble fusion form. Its ability to farnesylate ComX, which was generated by a ribosomally synthesized and post‐translationally modified peptide (RiPPs) approach, confirmed its activity and proper folding. Remarkably, TF‐ComQ_168_ also catalyzed farnesylation of daptomycin *in vitro*, most likely at the N‐terminal tryptophan residue. To explore substrate recognition, AlphaFold modeling was used to predict the binding pocket of ComQ_168_ with both ComX and daptomycin, highlighting a C‐terminal region potentially important for substrate interaction. These insights offer a promising starting point for engineering ComQ_168_ variants with expanded substrate scopes. In summary, this work demonstrates for the first time that ComQ_168_, previously known to modify linear peptides, can also farnesylate cyclic peptides—establishing its potential as a versatile tool in the biosynthetic engineering of bioactive compounds.

## Graphical abstract

ComQ_168_ from *Bacillus subtilis* 168 catalyzes C‐terminal tryptophan farnesylation of ComX to generate a competence‐inducing pheromone. Recombinant ComQ_168_ also modifies cyclic peptides, including Daptomycin, likely at the N‐terminal tryptophan. Structural modeling highlights a C‐terminal binding region, supporting substrate promiscuity and establishing ComQ_168_ as a versatile tool for engineering prenylated bioactive peptides.

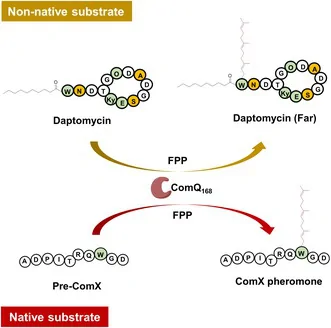

**Abbreviations**
FARM: first aspartate‐rich motif
FPP: farnesyl diphosphate
GPP: geranyl diphosphate
MALDI‐TOF: matrix‐assisted laser desorption ionization‐time‐of‐flight
NRPs: nonribosomal peptides
PTases: prenyltransferases
RiPPs: ribosomally synthesized and post‐translationally modified peptides
SARM: second aspartate‐rich motif
TF: trigger factor
Trp (W): tryptophan

## Introduction

Antibiotic resistance is a critical global health concern [1, 2, 3, 4]. Clinically important peptide antibiotics, such as daptomycin [5] and polymyxin [6, 7, 8], contain long fatty acid chains, which are attached by lipidation modification and play a key role in their antibacterial activities. Therefore, identifying and characterizing prenyltransferases (PTases) with broad substrate specificity that can catalyze lipidation modifications is essential for developing improved peptide antibiotic derivatives. In the process of peptide lipidation of the kingdom of bacteria, the PTases from cyanobacteria play a prominent role. These fascinating PTases are able to perform forward and reverse prenylation or geranylation, a special form of prenylation, on oxygen, nitrogen, and carbon atoms of tryptophan, serine, threonine, arginine, and histidine residues to produce various cyanobactins [9, 10, 11, 12, 13, 14, 15]. Apart from that, one PTase of particular interest is ComQ, involved in the biosynthesis of the *Bacillus subtilis* (*B. subtilis*) quorum‐sensing pheromone ComX.

The *comQXPA* gene cluster in *B. subtilis* regulates the development of genetic competence via secretion of the modified ComX pheromone [16, 17, 18]. In this system, *B. subtilis* synthesizes pre‐ComX, a linear peptide that is farnesylated by ComQ to produce the active ComX pheromone. When the concentration of this ComX pheromone reaches a threshold, it is sensed by ComP, which undergoes autophosphorylation via ATP and subsequently phosphorylates ComA. This activation cascade triggers downstream processes, such as surfactin biosynthesis, poly‐γ‐glutamate production, and the acquisition of genetic competence (Fig. 1). Clearly, ComQ‐mediated farnesylation is a critical step in this activation cascade. ComQ modifies an internal tryptophan residue within the ComX precursor peptide, resulting in a mature, farnesylated peptide essential for its role in competence signaling. Studying the substrate flexibility of ComQ will not only enhance our understanding of the *com*QXPA system but also unlock its potential as a tool enzyme for modifying antibiotic peptides—potentially leading to the development of more potent antimicrobial agents. Notably, ComQ not only modifies precursor peptides with long aliphatic chains but also exhibits the rare ability to generate complex geranylated or farnesylated tricyclic structures (Fig. S1) [19, 20, 21], which allows for the generation of a broader and more diverse structural library for downstream activity screening.

**Fig. 1:**
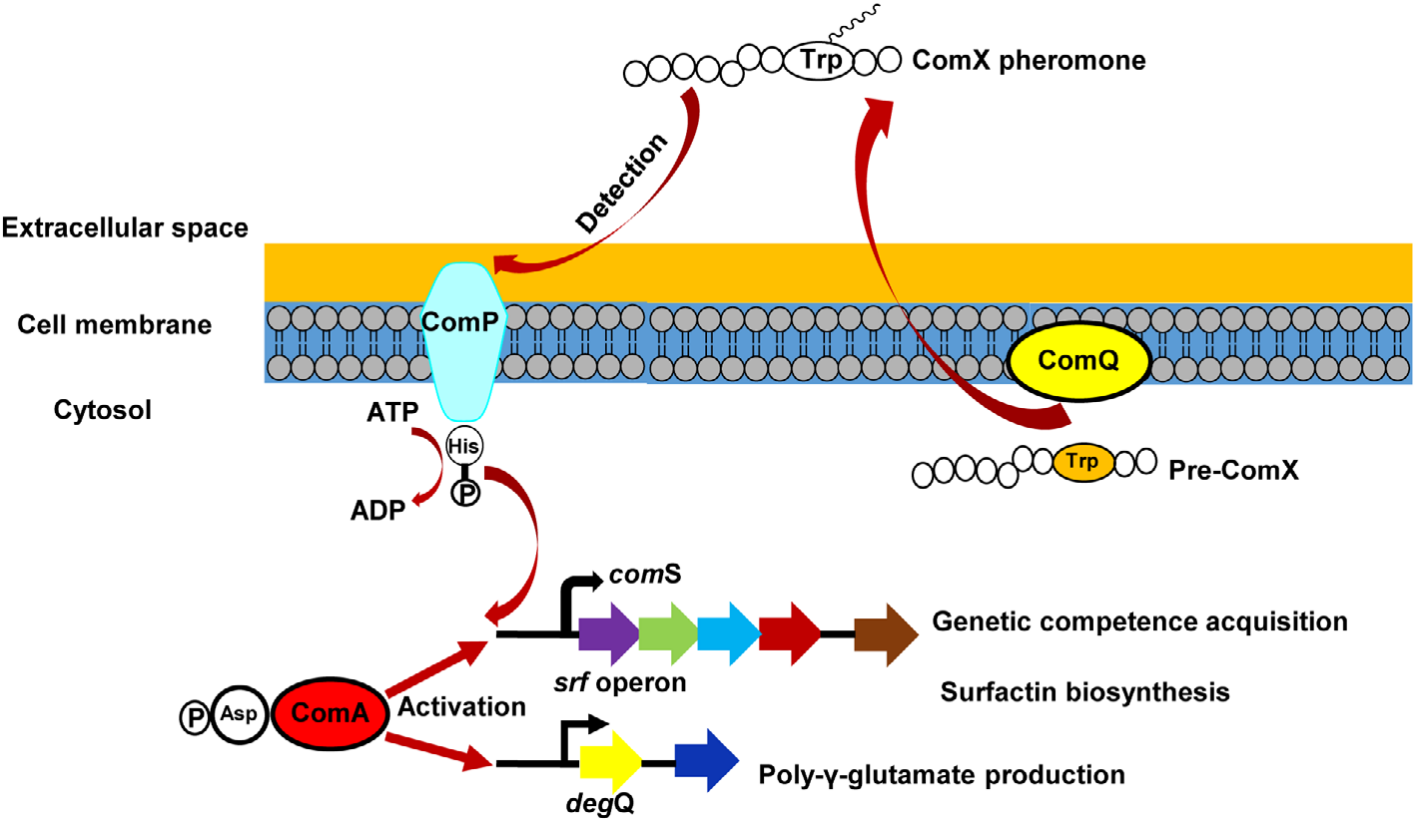
Scheme of quorum‐sensing responses through the ComQXPA system in *Bacillus subtilis*. Pre‐ComX is synthesized intracellularly, then undergoes lipidation, and is secreted outside the *Bacillus subtilis*. The ComP detects the lipidated ComX and becomes phosphorylated. Once activated, ComP transfers the phosphate group to ComA, initiating a cascade of intracellular signaling events. Tryptophan in pre‐ComX is highlighted in orange.

Extensive research on ComQ from *Bacillus* has been carried out. ComQs from different *Bacillus* species are aromatic prenyltransferases and contain two conserved aspartate‐rich motifs, known as the first aspartate‐rich motif (FARM) and the second aspartate‐rich motif (SARM) (Fig. S2). The ComQs are able to modify tryptophans (W) to form a tricyclic structure using farnesyl diphosphate (FPP) or geranyl diphosphate (GPP) as donor substrate, as shown in Fig. S1. When the donor substrate is GPP, the modification reaction is geranylation (C10) (Fig. S1). If it is FPP, it is called farnesylation (C15) (Fig. S1). Donor preferences of some prenyltransferases can be altered by site‐directed mutagenesis of amino acids in the active site [21, 22]. Generally, using a smaller amino acid in the acceptor active site to replace a larger one can be beneficial for accommodating longer donor sequences. This may be caused by the fact that small amino acids offer relatively more space in the active site. Among the various ComQs, ComQ_168_, ComQ_RO‐C‐2_, and ComQ_natto_ utilize FPP as a substrate donor for farnesylation, while ComQ_RO‐E‐2_, ComQ_RS‐B‐1_, and ComQ_RO‐B‐2_ use GPP for geranylation (Table S1). This suggests that the former group may possess a relatively larger active site to accommodate the longer donor substrate. Additionally, both ComQ_RO‐E‐2_ and ComQ_natto_ have been reported to modify a range of linear peptides, indicating that ComQ enzymes exhibit a certain degree of substrate flexibility [23, 24]. Taken together, these findings imply that ComQ_168_ may have a broad substrate tolerance. Based on this, ComQ_168_ was chosen as the focus of this study. The fatty acid of the marketed macrocyclic lipopeptide daptomycin is essential for its activity [25]. Lipidation of cyclic peptide substrates would greatly enhance the potential biochemical utilization of ComQ_168_, providing a vast potential for creating diverse molecules. More importantly, daptomycin contains an N‐terminal tryptophan, which is also quite different from ComQ_168_'s natural substrates considering the location of the tryptophan (Table S1). Therefore, daptomycin was taken as substrate to test the farnesylation potential of ComQ_168_ [26, 27, 28, 29].

Ribosomally synthesized and post‐translationally modified peptides (RiPPs) represent a broad and diverse class of complex natural peptides [30, 31]. Their biosynthetic principles can be exploited to generate not only a wide range of hybrid RiPPs [32] but also diverse nonribosomal peptide (NRP) variants [33], provided that the appropriate leader peptide and corresponding modification enzyme are available. In this study, as a first step, we successfully expressed and purified TF‐ComQ_168_ and produced its natural substrate ComX using a RiPP‐based system. Through successful farnesylation of ComX by TF‐ComQ_168_, we obtained active TF‐ComQ_168_. Subsequently, we tested the substrate tolerance of TF‐ComQ_168_ using the cyclic peptide daptomycin as a model substrate; we clearly proved for the first time that TF‐ComQ_168_ is able to modify cyclic peptides as substrates by demonstrating farnesylation, assumed to occur at the C‐3 position of tryptophan indole rings of the N‐terminal tryptophan of daptomycin. This greatly broadens the hitherto known limited substrate spectrum of ComQ_168_, which paves the way for the creation of numerous novel and active substances. Finally, initial modeling studies between ComQ_168_ and its natural substrate ComX yielded defined directions for subsequent ComQ_168_ engineering. Taken together, this study reveals the special substrate specificity of ComQ_168_ toward tryptophan and shows the possible feasibility to enzymatically farnesylated synthetic other cyclic peptides, which is a desirable feature considering that lipidation modification contributes a lot to antibiotic bioactivities.

## Results and Discussion

### Cloning, expression and purification of ComQ_168_

The first and most crucial step in studying ComQ_168_'s substrate tolerance is obtaining the ComQ_168_ itself. To achieve this, we first extracted the genomic DNA of *Bacillus subtilis* 168 according to materials and methods and used it as a template to obtain the ComQ_168_ gene. The primers used to amplify the ComQ_168_ gene fragment from the genome are shown in Table S2. The amplified ComQ_168_ encoding gene was 900 bp in length, which was verified by 1.2% agarose gels (Fig. S3), which is consistent with the theoretical length. Then, the ComQ_168_ gene was incorporated into the expression vector pCOLD‐TF, resulting in a recombinant expression plasmid pCOLD‐TF‐ComQ_168_, which was confirmed through DNA sequencing (Fig. 2A).

**Fig. 2:**
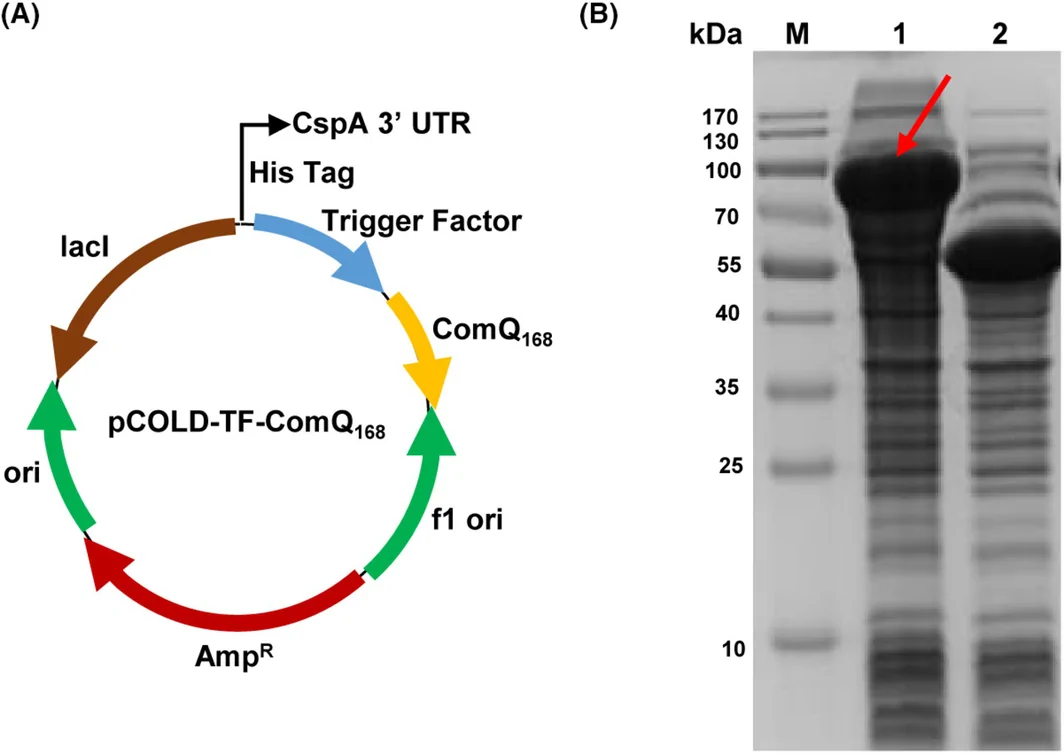
Results of the plasmid construction and heterologous expression. (A) Map of pCOLD‐TF‐ComQ_168_ plasmid for heterologous expression in *Escherichia coli* BL21(DE3). The pCold‐TF DNA vector is a fusion expression vector that expresses trigger factor (TF) chaperone as a soluble fusion tag. The pCold‐TF DNA vector is designed with a *cspA* promoter and several key downstream elements, including a 5′ untranslated region (5' UTR), a translation‐enhancing element (TEE), a His‐tag, and a multiple cloning site (MCS). To enable tight control over gene expression, a lac operator is positioned immediately downstream of the *cspA* promoter. Furthermore, the region between the MCS and the TF/His fusion tags include cleavage sites for HRV 3C Protease, Thrombin, and Factor Xa, allowing for efficient removal of the fusion tags from the target protein after expression. (B) Heterologous expression of the recombinant pCOLD‐TF‐ComQ_168_ in *E. coli* BL21(DE3). Protein markers indicated with kDa in the left margin. The red arrow indicated the band where trigger factor and ComQ_168_ protein were fused. (1) the crude extract expressing pCOLD‐TF‐ComQ_168_; (2) the crude extract expressing pCOLD‐TF as control.

Subsequently, plasmid pCOLD‐TF‐ComQ_168_ was introduced into *Escherichia coli* BL21(DE3) for heterologous expression. In parallel, *Escherichia coli* BL21(DE3) harboring pCOLD‐TF empty plasmid served as the control. After 20 h of culturing at 180 rpm and 15 °C, the soluble expression of TF‐ComQ_168_ was determined by SDS/PAGE analysis. As shown in Fig. 2B, Lane 1, an intense band of about 82.3 kDa was present in the crude extract containing pCOLD‐TF‐ComQ_168_, consistent with the mass of the expressed ComQ_168_ (34.3 kDa) fused with a trigger factor (TF) chaperone tag (48 kDa). Moreover, no corresponding band (Fig. 2B, lane 2) appeared in the controlled crude extract strain containing pCOLD‐TF, demonstrating that ComQ_168_ was successfully expressed as a fusion protein with a TF tag. After that, the expressed TF‐ComQ_168_ protein was purified using His‐Tag affinity chromatography. Proteins eluted with 150 mM imidazole were dialyzed and concentrated, and subsequently used as catalysts. (Fig. S4).

### Generation of native ComX by the RiPPs approach

Once the TF‐ComQ_168_ was obtained, a suitable substrate was needed to verify its activity. We chose ComX, the natural substrate of ComQ, to measure the activity of TF‐ComQ_168_. Various peptides are able to be generated as precursor peptides consisting of a leader peptide and a core peptide using the RiPPs biosynthetic pathway. We produced ComX using the RiPPs method (Fig. 3A) in this study. Specifically, the core peptide sequence of ComX was fused to a leader peptide, and the resulting construct was expressed in *E. coli* to generate the precursor peptide. After expression and purification, the peptide was analyzed using Tricine SDS/PAGE. As shown in Fig. 3B (indicated by the arrow), a clear band appeared around 10 kDa, which is close to the expected molecular weight and indicates a high yield.

**Fig. 3:**
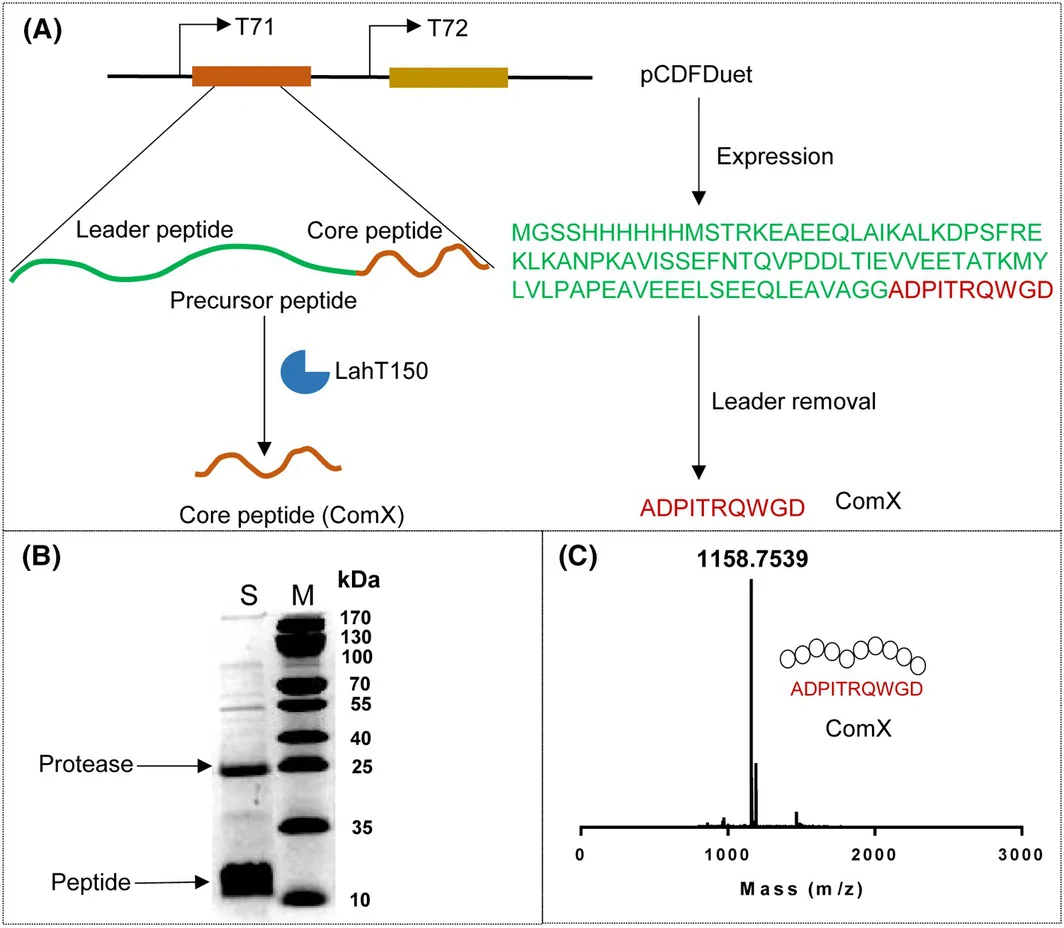
Production of ComX using the ribosomally synthesized and post‐translationally modified peptide (RiPPs) system and corresponding mass spectrometry analysis. (A) Schematic representation of ComX production via the RiPPs pathway. The precursor peptide is expressed under the T71 promoter of the pCDFDuet vector, and modifying enzymes are expressed under the T72 promoter. As ComX is a linear polypeptide without additional modifications, no modifying enzyme genes are present under the T72 promoter. The precursor peptide produced from T71 consists of a leader peptide and a core peptide. The green segment indicates the leader peptide, while the orange segment represents the core peptide, ComX. The enzyme LahT150 is used to specifically cleave the leader peptide. (B) Tricine SDS/PAGE analysis of the expressed precursor peptide. The arrow indicates the target band; S and M denote the sample and molecular weight marker, respectively. (C) MALDI‐TOF mass spectrometry analysis of the purified core peptide ComX, showing a molecular weight consistent with the predicted value.

Following this, the leader peptide was specifically cleaved using the LahT150 enzyme, which is a protease used to cleave the leader peptide, thereby releasing the core peptide [34] and the product was further purified using C18 chromatography. Mass spectrometry analysis confirmed successful production, with a detected molecular weight of 1158.75 Da, closely matching the predicted value of 1158.24 Da (Fig. 3C and Table S3). These results demonstrate that ComX can be efficiently produced in *E. coli* using the RiPPs system.

### ComQ_168_ activity assay

We successfully expressed and purified TF‐ComQ_168_ and generated its natural substrate ComX with RiPPs technologies in *E. coli*. Because TF‐ComQ_168_ was expressed as a fusion with the chaperone protein TF to facilitate proper folding, it was necessary to verify that the protein had adopted a functional conformation. We therefore assessed its enzymatic activity by examining its ability to catalyze the farnesylation of ComX. To achieve this, purified TF‐ComQ_168_ was incubated with ComX and FPP at 37 °C for 2 h in the presence of Mg^2+^. After centrifugation, the supernatant was dissolved in the 60% aqueous acetonitrile and analyzed by MALDI‐TOF‐MS. In the control reaction, an equal volume of MQ water was used in place of FPP, while all other conditions were kept identical. The results are presented in Fig. 4. As shown in Fig. 4, a distinct band corresponding to the expected molecular weight of ComX was observed, and mass spectrometry analysis further confirmed the successful production of the peptide. In the reaction group, a prominent peak showing a mass shift of approximately 204 Da—consistent with the expected modification—was detected (Table S3). Additionally, a clear difference between the chromatographic profiles of the control and reaction groups supports the conclusion that TF‐ComQ_168_ successfully modified ComX. This result not only confirms the enzymatic activity of ComQ_168_ but also provides a solid foundation for subsequent experiments and serves as a positive control.

**Fig. 4:**
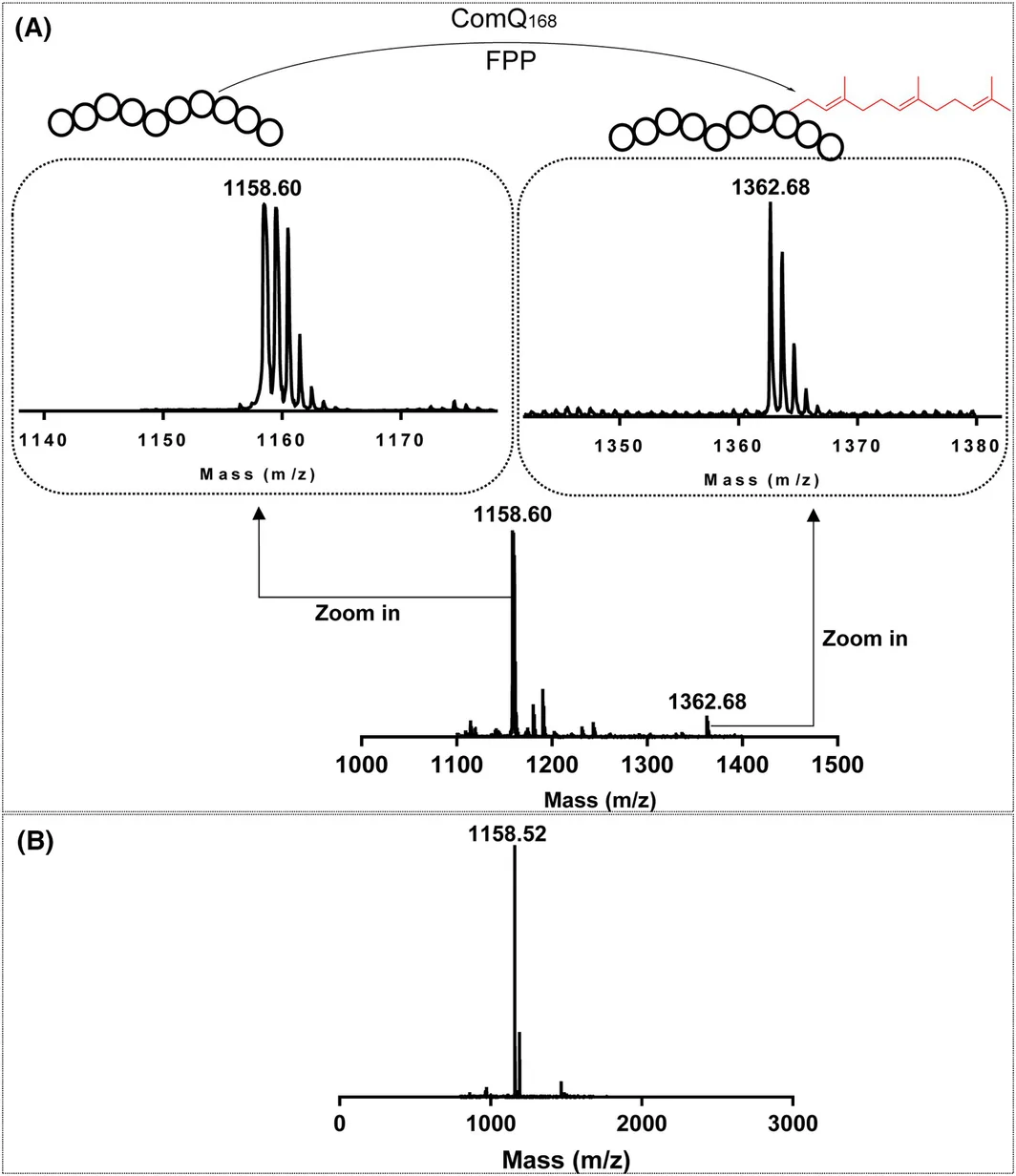
MALDI‐TOF‐MS analysis of the farnesylation of natural ComX by ComQ_168_. (A) MALDI‐TOF‐MS results showing the farnesylation of ComX catalyzed by ComQ_168_, along with a schematic of the farnesylation reaction and a zoomed‐in view of the mass spectrum. A distinct peak at 1362.6849 was observed in the reaction group, representing a mass shift of 204.09 Da relative to the unmodified ComX (1158.5956 Da), which closely matches the expected mass of a farnesyl group (204 Da). (B) MALDI‐TOF‐MS results from the control group, showing no corresponding mass shift, confirming that the modification is enzyme‐dependent.

### ComQ_168_ catalyzes farnesylation of daptomycin

Once a soluble, active form of the TF‐ComQ_168_ was successfully expressed and purified, the next step was to select an appropriate substrate to assess its substrate tolerance. Previous studies suggested that the length of the sequence between the tryptophan residue and the C terminus was important for geranylation by the ComQ_RO‐E‐2_ enzyme when employing various ComX_RO‐E‐2_ pheromone precursor analogs as substrates [20, 23]. In contrast, a more relaxed substrate specificity of ComQ_natto_, which catalyzes a unique farnesyl transfer reaction, has also been reported [24]. The substrate profile of ComQ_168_, derived from *Bacillus subtilis* 168, however, has not yet been experimentally evaluated. In this study, the commercial cyclic peptide daptomycin was used as a substrate to investigate the substrate tolerance of ComQ_168_.

For the farnesylation reaction, commercial daptomycin served as the substrate. The experimental setup involved incubating TF‐ComQ_168_ with the purified TF‐ComQ_168_ lysate and FPP in a buffer containing Mg^2+^ at 37 °C for 2 h. A parallel control reaction was conducted simultaneously, replacing TF‐ComQ_168_ with TF we purified from the empty plasmid pCOLD‐TF under identical conditions. After 2 h, the reactions were deemed complete. The control sample had remained clear, but, in contrast, the reaction mixture had become turbid, possibly indicating reduced solubility of the product (Fig. S5). We centrifuged the reaction mixture, extracted pellets with 60% aqueous acetonitrile and conducted MALDI‐TOF‐MS spectrometry. Mass spectrometry showed a new set of peaks (of 1824 Da and 1846 Da) in the reaction group compared with the control group (Fig. 5 and Table S3). We checked the molecular weight and found that the mass of the control peptide was 1619.96 Da and the mass of the new peak was 1824.46 Da, consistent with the calculated molecular weight after farnesylation (plus around 204 Da). In addition, MALDI‐TOF‐MS spectrometry analysis showed that the molecular weight of [M + Na]^+^ *m/z* 1643.19 and [M(Far) + Na]^+^ *m/z* 1846.44 corresponding to a daptomycin complex with one sodium ion (Table S4). This adduct is due to the presence of sodium ions in the reaction system. Taken together, the data obtained lead to the conclusion that ComQ_168_ modifies daptomycin. Previous research has demonstrated that prenyltransferases from other families, such as PagF [35] and LynF [9], consistently select tyrosine as modifying ligand, even when acting on non‐natural substrates. Similarly, for the homologous enzyme ComQ_168_, substrate spectrum analysis of ComQ_natto_ revealed that its catalytic site, like the wild‐type substrate, specifically targets tryptophan [24]. This suggests that in studies of substrate specificity for homologous enzymes and other prenyltransferases, the catalytic acceptor amino acid typically remains unchanged in the absence of enzyme engineering. Based on this observation, we hypothesize that the modification occurs on tryptophan. A structure after reaction is predicted and shown in Fig. S6A. This successful farnesylation is noteworthy for a few reasons: (a) contrary to ComQ's natural substrate, daptomycin is a cyclic peptide. (b) The length of the prenyl substrate accepted by ComQ_168_ is relatively rare among bacterial prenyltransferases, especially considering prenyltransferases from cyanobacteria, which tend to transfer DMAPP or GPP [9, 21, 35, 36, 37]. (c) Daptomycin's tryptophan is N‐terminally located and already bears a lipid group at the N‐terminal amino group. (d) We predict that the stereochemical configuration of the tryptophan residue modified by ComQ_168_ differs from that produced by bacterial prenyltransferases, such as KgpF [11].

**Fig. 5:**
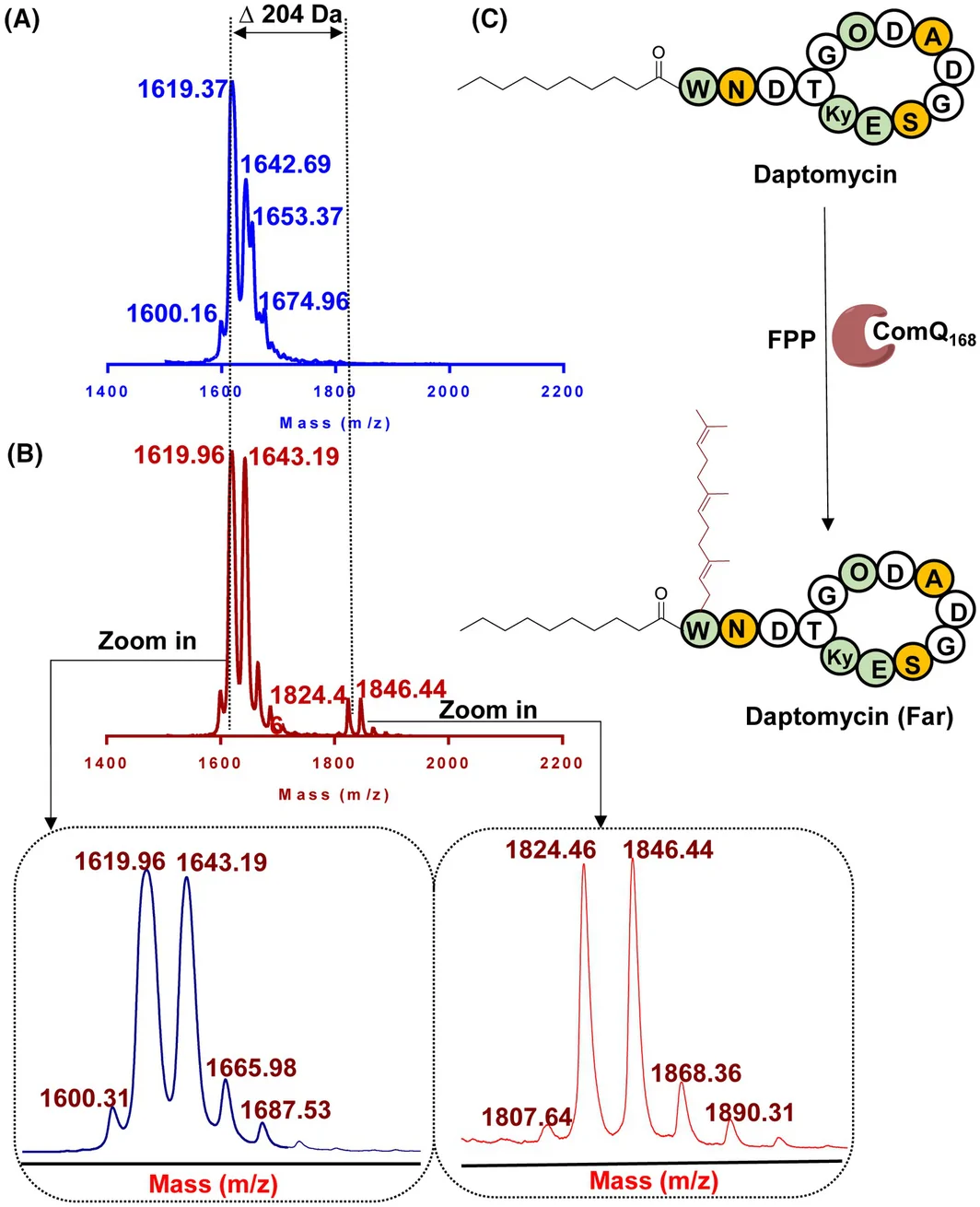
MALDI‐TOF‐MS analysis of farnesylation of daptomycin by ComQ_168_. (A) The blue spectrum represents the control group, with pCOLD‐TF plasmid encoded purified protein. (B) The upper dark brown spectrum represents the reaction group; the substrate is daptomycin, and the enzyme purified from the plasmid pCOLD‐TF‐ComQ_168_. The lower left spectrum represents amplified substrate daptomycin (blue color); the lower right spectrum represents amplified product prenylated daptomycin (red color). (C) The diagram of prenylation is shown on the right; the open black and white circles represent classical amino acids, the ochre circles represent D‐type amino acids, and the light green circles represent different types of nonclassical amino acids, where O represents ornithine, Ky represents kynurenine, E represents 3‐methyl‐glutamic acid. The ComQ_168_ was found to attach an additional tail on Trp, which is vertically depicted in red color.

ComQ_168_ modifies the C‐3 position of tryptophan indole rings of the precursor peptide ComX. It generates tricyclic scaffolds when using FPP as substrate, which ComQ_natto_ does (Fig. 1) [24]. However, the resulting stereochemistry of tricyclic scaffolds at the tryptophan C‐3 position is opposite to that of the KgpF, which is a cyanobacterial prenyltransferase with a broad substrate spectrum to linear and cyclic peptides [11, 38]. KgpF catalyzes stereospecific prenylation, leading to the formation of a tricyclic structure that shares the same core scaffold as that produced by ComQ_168_, but with opposite stereochemistry (Fig. S6). This stereochemical difference was confirmed through a combination of *in vitro* dimethylallylation of Fmoc‐tryptophan by KgpF and the chemical synthesis of dimethylallylated Fmoc‐tryptophan diastereomers [38]. Additionally, KgpF utilizes dimethylallyl pyrophosphate (DMAPP) as its prenyl donor and follows the same modification pattern as ComQ_168_. Although the natural substrates of ComQ_168_ and KgpF are vastly different, they both have the ability to prenylate or farnesylate C‐3 of Trp residues in both cyclic and linear peptides *in vitro*. The remarkable tolerance for peptide substrates of ComQ_168_ and KgpF showed unprecedented potential to create a vast structural diversity considering that they use different donor substrates and produce different product configurations.

### Structural modeling

Given the significant structural differences between daptomycin and ComQ_168_'s natural substrate, ComX, we performed molecular modeling of ComQ_168_ and its potential complexes to better understand their interaction. To investigate how ComQ_168_ interacts with daptomycin, we modeled ComQ_168_ using AlphaFold (AF) first due to the lack of an experimental structure. A FoldSeek search based on the AF model revealed many structurally similar proteins (average Cα RMSD: 1.8 Å), despite low sequence identity (< 20%) (Fig. 6A). Many searched proteins bound up to three Mg^2+^ ions, aligning with ComQ's known magnesium dependence. Then, we modeled ComQ_168_ with 1–3 Mg^2+^ ions using AF (Fig. S7A). All models were highly confident and consistently showed the C terminus blocking the active site. Further investigation and experimental verification of the functionality of the C terminus are needed.

**Fig. 6:**
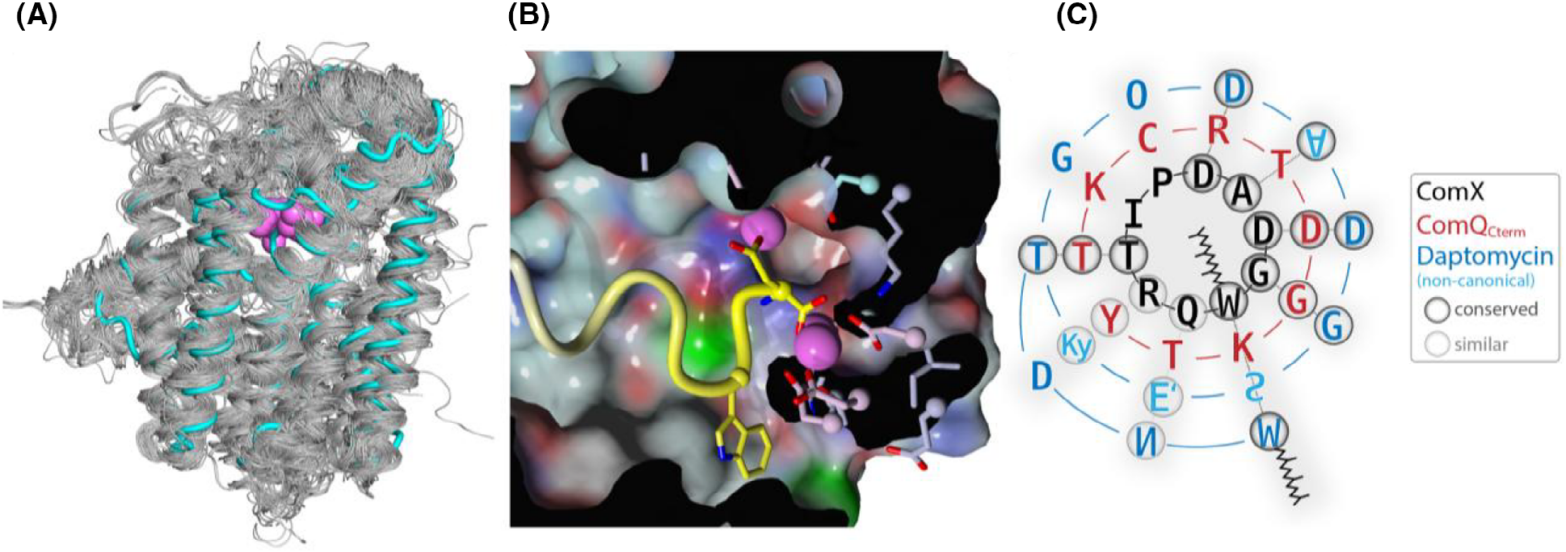
Structure search, modeling, and comparison of the peptides binding to ComQ_168_'s active site. Protein structure was predicted using AlphaFold Protein Structure Database, and structural similarity search was performed with Foldseek. (A) 150 top hits of a structural search of ComQ_168_ in the PDB databank aligned, ComQ_168_ is displayed in cyan. (B) A possible ComQ‐ComX pre‐catalytic complex as predicted by AF. Mg^2+^ in pink, ComX in yellow, and various active site residues of ComQ as well as ComX's terminal Asp as sticks. (C) Sequence alignment of the C terminus of ComQ_168_, daptomycin, and the natural core substrate ComX. In this figure, the inner ring (black) is ComX (ADPITRQWGD) with the modification on the W indicated. ‘Aligned’ on the second ring is the C terminus of ComQ (YTKGDTRCKT), where the cycle allows the C‐terminal T to continue alignment with the N‐terminal Y. In this alignment, 3/10 residues are conserved. The third ring is daptomycin's cyclic core, where seven residues are similar to the linear peptide, and three similar to ComQ‐C‐terminal. When aligning the linear N‐terminus as a fourth ring, even more similarities occur.

Next ComQ_168_ with its native substrate ComX was modeled. The C‐terminal region of ComX docked into ComQ_168_'s active site, replacing its own C terminus (Fig. S7B). One model placed the modified Trp near Mg^2+^, suggesting a plausible precatalytic complex (Fig. 6B). To simulate FPP binding, we modeled structures with adenosine diphosphate (ADP) and myristic acid. These mimics docked into a surface tunnel used for donor binding in homologs. Although a sequence alignment of the peptides did not show clear homology, we noticed that ComQ_168_'s C terminus features a Gly‐Asp motif that also occurs in ComX and, strikingly, that daptomycin contains Asp‐Gly (Fig. S8). Also, the Gly‐Asp motif is regarded as a critical substrate recognition motif in different peptides [39, 40]. Given that start and end, as well as the amino acid directionality is not clearly defined in cyclic peptides, and because several residues occur as D‐enantiomers, we figured that a similarity may only become apparent if we considered the reverse sequence, akin to retro‐inverso peptides [41]. Noticing further that AF modeled ComX in a conformation approaching a circle (Fig. S7C), accordingly we proposed a hypothesis and generated a ‘circular’ sequence alignment without defined start and end, and with both the forward and reverse sequence of daptomycin. Although not a clear ‘smoking gun’, we found a number of remarkable similarities (Fig. 6C). If indeed not a mere coincidence, these similarities suggest that daptomycin may adopt a position within ComQ similar to the one of ComX, with its cyclic core rotated such that the Trp binds in the catalytic center.

It is important to note that although our modeling results suggest possible mechanisms, the exact role of the C terminus of ComQ_168_ in catalysis and the detailed interaction between ComQ_168_ and daptomycin still need experimental validation. Nonetheless, these model data provide valuable insights and directions for future experiments.

In this study, we successfully biosynthesized the natural substrate ComX using the RiPPs strategy and expressed the tryptophan prenyltransferase ComQ_168_ from *Bacillus subtilis* 168 in *E. coli*. The ComQ_168_'s activity was first confirmed through its ability to modify ComX, and further demonstrated by its successful modification on the cyclic peptide daptomycin. This marks the first evidence that ComQ_168_ can modify cyclic peptides, expanding its known substrate scope beyond linear peptides. These results suggest that ComQ_168_ could serve as a promising tool for generating diverse daptomycin analogs and other modified bioactive peptides. Structural modeling of ComQ_168_ and its native substrate highlighted the potential regulatory role of its C terminus in catalysis. Based on these models, we propose that daptomycin may adopt a binding pose similar to ComX, offering new insights into ComQ_168_'s substrate recognition. Overall, these findings lay a foundation for future experimental validation and further exploration of ComQ_168_'s biocatalytic potential.

## Materials and methods

### Strains and materials

The strains *Escherichia coli* BL21(DE3) and *Bacillus subtilis* 168 were obtained from lab stocks from our Molecular Genetics Department of University of Groningen (https://www.molgenrug.nl/). Chemicals were purchased from Sigma unless indicated differently. Oligonucleotide primers were synthesized from Biolegio B.V. (Nijmegen, the Netherlands), and the purposes and sequences are listed in Table S2. The empty plasmid pCOLD‐TF was from lab stocks. The Gibson Assembly Master Mix enzyme was purchased from New England Biolabs (USA). Deoxynucleotides (dNTPs) and Phusion DNA Polymerase were purchased from Thermo Fisher Scientific (Waltham, MA, USA). Amplified DNA was purified using a NucleoSpin Gel and PCR Clean‐up Kit (Macherey‐Nagel). The plasmid DNA was isolated using a NucleoSpin Plasmid EasyPure Kit (Macherey‐Nagel). The pCOLD‐TF‐ComQ_168_ plasmid sequences were confirmed by Macrogen Europe (Amsterdam, the Netherlands) with specific sequencing primers (Table S2).

### Plasmid construction

The sequence coding for ComQ_168_ was retrieved from SubtiWiki (http://subtiwiki.uni‐goettingen.de/). The base and amino acid sequences of ComQ_168_ are shown in the Supporting Information. The primers are designed and synthesized based on the Gibson assembly method. The sequence coding for the ComQ_168_ was amplified by polymerase chain reaction (PCR) from the genome of *Bacillus subtilis* 168, which was extracted according to the standard bacterial DNAextraction Kit. The amplified ComQ_168_ gene was confirmed by 1.2% agarose gel electrophoretic analyses (Fig. S2). Amplified DNA was cleaned by a NucleoSpin Gel and the PCR Clean‐up Extraction Kit (Macherey–Nagel). The vector part (pCOLD‐TF) can be amplified and purified by the same methods. The Gibson Assembly ligation reaction was performed according to the manufacturer's instructions. The ligation products were transformed to *E. coli* TOP10 cells by chemical transformation. Cells were plated on Lysogeny Broth (LB) agar plates with 10 μg·ml^−1^ of ampicillin antibiotics and grown at 37 °C overnight. Several transformants were picked and inoculated into LB medium with the appropriate amount of ampicillin. The cultures were grown at 37 °C overnight, and plasmids were isolated using a NucleoSpin Plasmid Extraction Kit (Macherey–Nagel), and the desired constructs were confirmed by sequencing.

### Heterologous expression and preparation of ComQ_168_

The expression plasmid pCOLD‐TF‐ComQ_168_ was introduced into *E. coli* BL21(DE3) for heterologous expression by chemical transformation. One single colony was inoculated in 5‐mL LB broth medium with 10 μg·mL^−1^ of ampicillin antibiotic to culture overnight at 37 °C with 220 rpm. Two milliliters of overnight culture was added into 100 mL fresh LB broth medium (1 : 50), resulting a diluted and refreshed culture, which was induced by the addition of IPTG to a final concentration of 0.5 mm when OD_600_ value reached around 0.6. The culture was continued induction for 24 h at 15 °C. Next, the induced cells were harvested by centrifugation at 10000 rpm for 5 min and the supernatant is discarded. The pellets were resuspended in PBS buffer (20 mm, pH = 8.0). The suspended cells were disrupted by sonication for 15 working minutes with an amplitude of 45% (10 s on and 10 s off). The lysate was obtained by centrifugation at 10 000 rpm for 15 min. The supernatant was filtered through 0.45 μm filters. The sample was subject to His‐Tag affinity purification using a Ni‐NTA agarose column. Different concentrations of imidazole from 20 mm to 150 mm are used to elute to obtain different fractions which were confirmed by SDS/PAGE. Finally, the fractions containing the target protein was further purified and concentrated by a 50 mL Amicon® Ultra‐50 tube. The control group was the same as above described except that the introduced plasmid was empty pCOLD‐TF in the first day and the tube used for final concentration was Amicon^®^ Ultra‐30.

### Expression and purification of ComX from *Escherichia coli*

The pCDFDuet‐ComX‐core plasmid was transformed into *E. coli* BL21(DE3) competent cells and plated on LB agar containing spectinomycin. Single colonies were cultured overnight in 5 mL LB broth with 50 μg·mL^−1^ spectinomycin at 37 °C and 220 rpm. The overnight culture was then diluted 1 : 50 into Terrific Broth (TB) supplemented with spectinomycin (50 μg·mL^−1^) and grown at 37 °C until reaching an OD₆₀₀ of 1.0–2.0. Cultures were chilled on ice, induced with 1 mm IPTG, and incubated at 18 °C for ~ 20 h at 200 rpm for peptide expression.

Cells from 100 mL of culture were harvested by centrifugation (4 °C, 8500 rpm, 5 min), resuspended in lysis buffer (20 mm NaH_2_PO_4_, 300 mm NaCl, 10 mm imidazole, pH 7.4), and lysed via sonication (10 s on/off, 45–55% amplitude, 10–15 min). The lysate was clarified by centrifugation (4 °C, 10 000 rpm, 30 min) and filtered (0.45 μm). The supernatant was applied to a Ni‐NTA column, washed with 10 column volumes (CV) of wash buffer (40 mm imidazole), and eluted with 500 mM imidazole.

The eluted sample was desalted using a PD‐10 column equilibrated with 50 mm Tris–HCl (pH 8.0). To cleave the leader peptide, LahT150 protease (1 : 20 ratio, 1 mm DTT) was added, and the mixture was incubated at 37 °C for 2 h. After centrifugation (4 °C, 8000 ***g***, 15 min) and filtration (0.45 μm), the mixture was passed through a second Ni‐NTA column. The flow‐through, containing the cleaved core peptide, was collected and buffer exchanged with 20 mm Tris, 300 mM NaCl (pH 7.5).

For final purification, the core peptide was loaded onto a C18 open column, washed with 0.1% TFA in acetonitrile (ACN), equilibrated with 0.1% TFA in Milli‐Q water, and eluted using 60% ACN + 0.1% TFA. The eluate was lyophilized, and the purified ComX core peptide was used for downstream analysis.

### Farnesylation assays *in vitro*

The purified ComQ_168_ was used as the biocatalyst for farnesylation assays. In a final volume of 100 μL, 5 mm daptomycin, 5 mm farnesyl pyrophosphate ammonium salt (Sigma‐Aldrich), and concentrated ComQ_168_ were incubated for 2 h at 37 °C in pH = 8.0, 20 mm PBS buffer containing 5 mm MgCl_2_ and 5 mm CaCl_2_. In the control group, the product purified from empty pCOLD‐TF plasmid was used instead of ComQ_168_, that is, we purified trigger factor carrying his‐tag as a control and other conditions were the same. After 2 h, the reaction was stopped by centrifugation at 10 000 ***g*** for 10 min. The supernatant was transferred to another tube and addition of 50 μL of 60% aqueous acetonitrile to resuspend the pellet and centrifuge again. The supernatant was subject to MALDI‐TOF‐MS analysis.

### MALDI‐TOF mass spectrometry

For MALDI‐TOF analysis, 0.5 μL sample was spotted and dried on the target. Subsequently, 0.5 μL of matrix solution (5 mg·mL^−1^ α‐cyano‐4‐hydroxycinnamic acid from Sigma‐Aldrich dissolved in 50% aqueous acetonitrile containing 0.1% trifluoroacetic acid) was spotted on top of the sample. MALDI‐TOF‐MS analyses were carried out using a 4800 Plus MALDI TOF/TOF Analyzer (Applied Biosystems, Waltham, MA, USA). Data were acquired in linear positive‐ion mode for samples containing daptomycin and in reflector mode for samples containing ComX.

### Computational modeling

Computational models of ComQ_168_ in complex with magnesium and ComX were generated using AlphaFold3 as implemented on https://alphafoldserver.com [42]. Structural homologs in the PDB and the AF database were identified with FoldSeek using the command line version from https://github.com/steineggerlab/foldseek [43]. Structural alignments and visualizations were performed with YASARA [44], all sequence alignments were done with ClustalW.

## Author contributions

YX and OPK conceived the project and strategy. YX designed and carried out the experiments, analyzed data, and wrote the manuscript. MJLJF performed the docking analysis, contributed to the initial draft of the docking section, and corrected the manuscript. OPK supervised and corrected the manuscript. All authors contributed to and commented on the manuscript text and approved its final version.

## Conflicts of interest

The authors declare no conflict of interest.

## Supporting information


**Table S1.** Amino acid sequences and modification type of different *Bacillus* strains.
**Table S2**. Primer sequences used in this study.
**Table S3**. Summary of observed and calculated MALDI‐TOF MS data in this study ([M + H]^+^).
**Table S4**. Summary of observed and calculated MALDI‐TOF MS data in this study ([M + Na]^+^).
**Fig. S1**. Prenyltransferase‐modified Trp residue of ComX pheromones. The structure of the prenyltransferase‐modified Trp residue of ComX pheromones. Chemical structures of the Trp residue before (middle), after ComQ‐mediated geranylation (left) and after ComQ‐mediated farnesylation (right). The blue and purple crescents represent ComQ from different *Bacillus* species. Blue is related to farnesylation, while purple is involved in geranylation.
**Fig. S2**. Comparison of amino acid sequences of ComQ from different Bacillus species. The accession numbers of the prepursor ComXs from different *bacillus* species are AAL67728 (RO‐C 2), AIY94492 (168), AAL67740 (RO‐E‐2), AAF82173 (RS‐B‐1), and AAL67731 (RO‐B‐2). These enzymes contain two conserved aspartate‐rich motifs, known as the first aspartate‐rich motif (FARM) and the second aspartate‐rich motif (SARM), highlighted in the red box of the Figure. The FARM motif in ComQ is highly conserved, whereas the residues corresponding to the SARM motif show considerable variation. Multiple sequence alignment was performed using ClustalW and visualized using ESPript.
**Fig. S3**. 1.2% TAE agarose gel electrophoretic analyses for ComQ_168_ amplification. M: 1 kb DNA ladder; S: amplified ComQ168 sample. The ComQ_168_ gene is 900 bp in length. The nucleotide sequence is provided below.
**Fig. S4**. Different concentrations of imidazole elution solution for concentrating pCOLD‐TF ComQ168. Numbers from 20 to 150 represent different concentrations of imidazole (mM). ComQ_168_ has an expected molecular weight of ~34 kDa, and the Trigger Factor (TF) chaperone is ~48 kDa. The TF ComQ_168_ fusion (target) protein is approximately 82 kDa. The red arrow indicates the target protein band.
**Fig. S5**. Visual comparison of the *in vitro* farnesylation reaction and the control reaction. After incubation, the reaction mixture containing ComQ_168_ appeared turbid, whereas the control remained relatively clear. The control sample is shown on the left, and the reaction sample on the right. Each reaction had a total volume of 100 μL. The reaction mixture contained 5 mM MgCl_₂_, 5 mM CaCl_₂_, 5 mM daptomycin, 5 mM farnesyl pyrophosphate ammonium salt, and concentrated ComQ168 in 20 mM PBS buffer (pH 8.0). In the control reaction, the empty pCOLD‐TF plasmid was used in place of ComQ_168_.
**Fig. S6**. Chemical structure of the modified tryptophan residues by ComQ_168_ and KgpF. Both enzymes generate a similar tricyclic structure as part of their modification, highlighting a shared pattern. However, key differences are also present and are color‐coded in the Figure: red indicates differences in the required prenyl donors, while green highlights variations in the stereochemistry structures of the resulting products. (A) modified by ComQ168; (B) modified by KgpF.
**Fig. S7**. Structural simulation analyses of the ComQ_168_ and its homologous enzymes. Protein structure was predicted using AlphaFold Protein Structure Database. (A) ComQ apo interaction of 2 Mg^2+^ with C‐terminal carboxy. (B) AF3 models of ComQ in complex with short ComX, in which ComX always displaces ComQ's C terminus from the active site. (C) AF3 places ComX in the active site in a conformation approximating a circle.
**Fig. S8**. The chemical structure of daptomycin is depicted, with the corresponding amino acids labeled in red. The D amino acid, which were present in blue in the Figure. The DG motif, identical to the C terminus of ComQ, is highlighted in yellow and the background is also in yellow. Ky: kynurenine, O: ornithine.

## Data Availability

The data that support the findings of this study are available from the corresponding author [o.p.kuipers@rug.nl] upon reasonable request.

## References

[1] Marston HD , Dixon DM , Knisely JM , Palmore TN & Fauci AS (2016) Antimicrobial resistance. JAMA J Am Med Assoc 316, 1193–1204. 10.1001/jama.2016.11764 27654605

[2] Tang KWK , Millar BC & Moore JE (2023) Antimicrobial Resistance (AMR). Br J Biomed Sci 80, 11387. 10.3389/bjbs.2023.11387 PMC10336207 37448857

[3] Hou J , Long X , Wang X , Li L , Mao D , Luo Y & Ren H (2023) Global trend of antimicrobial resistance in common bacterial pathogens in response to antibiotic consumption. J Hazard Mater 442, 130042. 10.1016/j.jhazmat.2022.130042 36182890

[4] Garg R (2024) A review on antibiotic resistance. Indian J Health Care Med Pharm Pract 5, 50–56.

[5] Heidary M , Khosravi AD , Khoshnood S , Nasiri MJ , Soleimani S & Goudarzi M (2018) Daptomycin. J Antimicrob Chemother 73, 1–11. 10.1093/jac/dkx349 29059358

[6] Slingerland CJ & Martin NI (2023) Recent advances in the development of polymyxin antibiotics: 2010–2023. ACS Infect Dis 10, 1056–1079. 10.1021/acsinfecdis.3c00630 PMC11019560 38470446

[7] Arnold TM , Forrest GN & Messmer KJ (2007) Polymyxin antibiotics for gram‐negative infections. Am J Heal Pharm 64, 819–826. 10.2146/ajhp060473 17420197

[8] Velkov T , Thompson PE , Nation RL & Li J (2010) Structure‐activity relationships of polymyxin antibiotics. J Med Chem 53, 1898–1916. 10.1021/jm900999h PMC2907661 19874036

[9] McIntosh JA , Donia MS , Nair SK & Schmidt EW (2011) Enzymatic basis of ribosomal peptide prenylation in cyanobacteria. J Am Chem Soc 133, 13698–13705. 10.1021/ja205458h PMC3170831 21766822

[10] Okada M , Sugita T & Abe I (2017) Posttranslational isoprenylation of tryptophan in bacteria. Beilstein J Org Chem 13, 338–346. 10.3762/bjoc.13.37 PMC5331326 28326143

[11] Ishida K , Matsuda H , Murakami M & Yamaguchi K (1997) Kawaguchipeptin B, an antibacterial cyclic undecapeptide from the cyanobacterium Microcystis aeruginosa. J Nat Prod 60, 724–726. 10.1021/np970146k 9249979

[12] Morita M , Hao Y , Jokela JK , Sardar D , Lin Z , Sivonen K , Nair SK & Schmidt EW (2018) Post‐translational tyrosine geranylation in cyanobactin biosynthesis. J Am Chem Soc 140, 6044–6048. 10.1021/jacs.8b03137 PMC6242345 29701961

[13] Parajuli A , Kwak DH , Dalponte L , Leikoski N , Galica T , Umeobika U , Trembleau L , Bent A , Sivonen K , Wahlsten M *et al*. (2016) A unique tryptophan C‐Prenyltransferase from the Kawaguchipeptin biosynthetic pathway. Angew Chem 55, 3596–3599. 10.1002/anie.201509920 PMC5108402 26846478

[14] Zhang Y , Hamada K , Thanh Nguyen D , Inoue S , Satake M , Kobayashi S , Okada C , Ogata K , Okada M , Sengoku T *et al*. (2022) LimF is a versatile prenyltransferase catalyzing histidine‐C‐geranylation on diverse nonnatural substrates. Nat Catal 5, 682–693.

[15] Phan CS , Matsuda K , Balloo N , Fujita K , Wakimoto T & Okino T (2021) Argicyclamides A‐C unveil enzymatic basis for guanidine bis‐prenylation. J Am Chem Soc 143, 10083–10087. 10.1021/jacs.1c05732 34181406

[16] Magnuson R , Solomon J & Grossman AD (1994) Biochemical and genetic characterization of a competence pheromone from B. Subtilis. Cell 77, 207–216. 10.1016/0092-8674(94)90313-1 8168130

[17] Schneider KB , Palmer TM & Grossman AD (2002) Characterization of comQ and comX, two genes required for production of comX pheromone in Bacillus subtilis. J Bacteriol 184, 410–419. 10.1128/JB.184.2.410-419.2002 PMC139578 11751817

[18] Ansaldi M , Marolt D , Stebe T , Mandic‐Mulec I & Dubnau D (2002) Specific activation of the bacillus quorum‐sensing systems by isoprenylated pheromone variants. Mol Microbiol 44, 1561–1573. 10.1046/j.1365-2958.2002.02977.x 12067344

[19] Okada M , Sato I , Cho SJ , Iwata H , Nishio T , Dubnau D & Sakagami Y (2005) Structure of the bacillus subtilis quorum‐sensing peptide pheromone Comx. Nat Chem Biol 1, 23–24. 10.1038/nchembio709 16407988

[20] Tsuji F , Ishihara A , Nakagawa A , Okada M , Kitamura S , Kanamaru K , Masuda Y , Murakami K , Irie K & Sakagami Y (2012) Lack of the consensus sequence necessary for tryptophan prenylation in the ComX pheromone precursor. Biosci Biotechnol Biochem 76, 1492–1496. 10.1271/bbb.120206 22878193

[21] Estrada P , Morita M , Hao Y , Schmidt EW & Nair SK (2018) A single amino acid switch alters the isoprene donor specificity in Ribosomally synthesized and post‐translationally modified peptide Prenyltransferases. J Am Chem Soc 140, 8124–8127. 10.1021/jacs.8b05187 PMC6467259 29924593

[22] Hirooka K , Shioda S & Okada M (2020) Identification of critical residues for the catalytic activity of ComQ, a bacillus prenylation enzyme for quorum sensing, by using a simple bioassay system. Biosci Biotechnol Biochem 84, 347–357. 10.1080/09168451.2019.1685371 31670609

[23] Tsuji F , Ishihara A , Kurata K , Nakagawa A , Okada M , Kitamura S , Kanamaru K , Masuda Y , Murakami K , Irie K *et al*. (2012) Geranyl modification on the tryptophan residue of ComX RO‐E‐2 pheromone by a cell‐free system. FEBS Lett 586, 174–179. 10.1016/j.febslet.2011.12.012 22197102

[24] Sugita T , Okada M , Nakashima Y , Tian T & Abe I (2018) A tryptophan prenyltransferase with broad substrate tolerance from Bacillus subtilis subsp. natto. Chembiochem 19, 1396–1399. 10.1002/cbic.201800174 29665236

[25] Chow HY , Po KHL , Jin K , Qiao G , Sun Z , Ma W , Ye X , Zhou N , Chen S & Li X (2020) Establishing the structure‐activity relationship of daptomycin. ACS Med Chem Lett 11, 1442–1449. 10.1021/acsmedchemlett.0c00175 PMC7357220 32676152

[26] Scull EM , Bandari C , Johnson BP , Gardner ED , Tonelli M , You J , Cichewicz RH & Singh S (2020) Chemoenzymatic synthesis of daptomycin analogs active against daptomycin‐resistant strains. Appl Microbiol Biotechnol 104, 7853–7865. 10.1007/s00253-020-10790-x PMC7447621 32725322

[27] Li X (2022) Long journey on daptomycin. Synlett 33, 27–33.

[28] Mupparapu N , Lin YHC , Kim TH & Elshahawi SI (2021) Regiospecific synthesis of calcium‐independent daptomycin antibiotics using a chemoenzymatic method. Chem Eur J 27, 4176–4182. 10.1002/chem.202005100 33244806

[29] Barnawi G , Noden M , Goodyear J , Marlyn J , Schneider O , Beriashvili D , Schulz S , Moreira R , Palmer M & Taylor SD (2022) Discovery of highly active derivatives of daptomycin by assessing the effect of amino acid substitutions at positions 8 and 11 on a daptomycin analogue. ACS Infect Dis 8, 778–789. 10.1021/acsinfecdis.1c00483 35317552

[30] Han SW & Won HS (2024) Advancements in the application of Ribosomally synthesized and post‐translationally modified peptides (RiPPs). Biomolecules 14, 479. 10.3390/biom14040479 PMC11048544 38672495

[31] Arnison PG , Bibb MJ , Bierbaum G , Bowers AA , Bugni TS , Bulaj G , Camarero JA , Campopiano DJ , Challis GL , Clardy J *et al*. (2013) Ribosomally synthesized and post‐translationally modified peptide natural products: overview and recommendations for a universal nomenclature. Nat Prod Rep 30, 108–160. 10.1039/c2np20085f PMC3954855 23165928

[32] Xu Y , Reuvekamp R & Kuipers OP (2024) Biosynthesis of antimicrobial ornithine‐containing Lacticin 481 analogues by use of a combinatorial biosynthetic pathway in Escherichia coli. ACS Synth Biol 13, 4209–4217. 10.1021/acssynbio.4c00650 PMC11669161 39660664

[33] Xu Y , Ruijne F , Diez MG , Stada JJ & Kuipers OP (2025) Design and production of geranylated cyclic peptides by the RiPP enzymes SyncM and PirF. Biomacromolecules 26, 3186–3199. 10.1021/acs.biomac.5c00260 PMC12076493 40189806

[34] Bobeica SC , Dong SH , Huo L , Mazo N , McLaughlin MI , Jiménez‐Osés G , Nair SK & van der Donk WA (2019) Insights into AMS/PCAT transporters from biochemical and structural characterization of a double glycine motif protease. elife 8, e42305. 10.7554/eLife.42305 PMC6363468 30638446

[35] Hao Y , Pierce E , Roe D , Morita M , McIntosh JA , Agarwal V , Cheatham TE , Schmidt EW & Nair SK (2016) Molecular basis for the broad substrate selectivity of a peptide prenyltransferase. Proc Natl Acad Sci USA 113, 14037–14042. 10.1073/pnas.1609869113 PMC5150373 27872314

[36] Martins J , Leikoski N , Wahlsten M , Azevedo J , Antunes J , Jokela J , Sivonen K , Vasconcelos V , Fewer DP & Leão PN (2018) Sphaerocyclamide, a prenylated cyanobactin from the cyanobacterium Sphaerospermopsis sp. LEGE 00249. Sci Rep 8, 6831. 10.1038/s41598-018-32618-5 PMC6162287 30266955

[37] Dalponte L , Parajuli A , Younger E , Mattila A , Jokela J , Wahlsten M , Leikoski N , Sivonen K , Jarmusch SA , Houssen WE *et al*. (2018) N‐prenylation of tryptophan by an aromatic Prenyltransferase from the cyanobactin biosynthetic pathway. Biochemistry 57, 6860–6867. 10.1021/acs.biochem.8b00879 30452235

[38] Okada M , Sugita T , Akita K , Nakashima Y , Tian T , Li C , Mori T & Abe I (2016) Stereospecific prenylation of tryptophan by a cyanobacterial post‐translational modification enzyme. Org Biomol Chem 14, 9639–9644. 10.1039/c6ob01759b 27714214

[39] Hover BM , Kim SH , Katz M , Charlop‐Powers Z , Owen JG , Ternei MA , Maniko J , Estrela AB , Molina H , Park S *et al*. (2018) Culture‐independent discovery of the malacidins as calcium‐dependent antibiotics with activity against multidrug‐resistant gram‐positive pathogens. Nat Microbiol 3, 415–422. 10.1038/s41564-018-0110-1 PMC5874163 29434326

[40] Zhu Z , Zhang B , Cai Q , Cao Y , Ling J , Lee K & Chen B (2021) A critical review on the environmental application of lipopeptide micelles. Bioresour Technol 339, 125602. 10.1016/j.biortech.2021.125602 34311406

[41] Okada M (2011) Post‐translational isoprenylation of tryptophan. Biosci Biotechnol Biochem 75, 1413–1417. 10.1271/bbb.110087 21821957

[42] Abramson J , Adler J , Dunger J , Evans R , Green T , Pritzel A , Ronneberger O , Willmore L , Ballard AJ , Bambrick J *et al*. (2024) Accurate structure prediction of biomolecular interactions with AlphaFold 3. Nature 630, 493–500. 10.1038/s41586-024-07487-w PMC11168924 38718835

[43] van Kempen M , Kim SS , Tumescheit C , Mirdita M , Lee J , Gilchrist CLM , Söding J & Steinegger M (2024) Fast and accurate protein structure search with Foldseek. Nat Biotechnol 42, 243–246. 10.1038/s41587-023-01773-0 PMC10869269 37156916

[44] Ozvoldik K , Stockner T & Krieger E (2023) YASARA model‐interactive molecular modeling from two dimensions to virtual realities. J Chem Inf Model 63, 6177–6182. 10.1021/acs.jcim.3c01136 PMC10598798 37782001

