# Supplementary material for: The tryptophan prenyltransferase ComQ from Bacillus subtilis 168 can prenylate daptomycin at Trp1
Source: FEBS J. 2026 May 2;293(21):6413–25. doi: 10.1111/febs.70578 (PMC13731918; doi:10.1111/febs.70578)
Supplement: Supplementary file 1 [file FEBS-293-6413-s001.pdf]

SUPPORTING INFORMATION

The Tryptophan Prenyltransferase ComQ from *Bacillus subtilis* 168 can Prenylate Daptomycin at Trp1

Yanli Xu[1], Maximilian J.L.J. Fürst[2], Oscar P. Kuipers[1,*]

[1]Department of Molecular Genetics, Groningen Biomolecular Sciences and Biotechnology Institute, University of Groningen, Groningen, 9747 AG, The Netherlands.

[2]Molecular Enzymology, Groningen Biomolecular Sciences and Biotechnology Institute, University of Groningen, Feringa Building, Groningen, 9747 AG, The Netherlands.

***** Correspondence: Oscar P. Kuipers; E-mail: o.p.kuipers@rug.nl ; Tel: +31 50 3632093.

Table S1 Amino acid sequences and modification type of different *Bacillus* strains.

***Bacillus* strain**	**ComX pheromone**	**Modification Type**	**ComQ**
168	ADPITRQ**W**GD	Farnesylation	
Natto	K**W**PPIE	Farnesylation	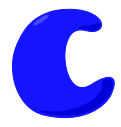
RO-C-2	TRE**W**DG	Farnesylation	
RO-E-2	GIF**W**EQ	Geranylation	
RS-B-1	MMD**W**HY	Geranylation	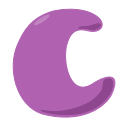
RO-B-2	YTNGN**W**VPS	Geranylation	

Amino acid sequences and modification type of different *Bacillus* strains are presented in the table. Modified Trp residues in the ComX pheromone sequence are in bold. The blue and purple crescents represent ComQ from different *Bacillus* species. Blue is related to farnesylation modification while purple is involved in geranylation modification.

Table S2 Primer sequences used in this study.

**Primer**	**Sequences (5' to 3')**	**Purpose**
ComQ-F	ATGAAGGAGATTGTGGAGCAAAACA	Forward Primer for ComQ gene amplification (pCOLD-TF- ComQ$_{168}$)
ComQ-R	TTAGGTCTTGCATCTTGTATCCCCC	Reverse Primer for ComQ gene amplification (pCOLD-TF- ComQ$_{168}$)
ComQ-pCOLD-TF-F	GGGGGATACAAGATGCAAGACCTAAGAG CTCGGTACCCTCGAG	Forward Primer for pCOLDTF vector amplification (pCOLD-TF- ComQ$_{168}$)
ComQ-pCOLD-TF-R	TGTTTTGCTCCACAATCTCCTTCATATGC CTACCTTCGATACCACC	Reverse Primer for pCOLDTF vector amplification (pCOLD-TF- ComQ$_{168}$)
Linear natural ComX core-Fw	GATCACCCGTCAGTGGGGTGACTAAGCT TGCGGCCGCATA ATGCTTAAGTCG	Construction of pCDFDuet-ComX-core
Linear natural ComX core Rv	CCCCACTGACGGGTGATCGGGTCAGCG CCACCGCTGGCTG CTTCCAGTTC	Construction of pCDFDuet-ComX-core
pCOLD-TF-Fw-sequencing	CTGCGTACGAAGATCCGAAAG	Sequencing primer for pCOLD-TF-ComQ$_{168}$
pCOLD-TF-Rv-sequencing	GGCAGGGATCTTAGATTCTG	Sequencing primer for pCOLD-TF-ComQ$_{168}$

Table S3 Summary of observed and calculated MALDI–TOF MS data in this study(**[M+H]$^+$**).

**Name**	**m/z [M+H]$^+$** (calculated) **(Da)**	**m/z [M+H]$^+$** (observed) **(Da)**
ComX	1158.55	1158.75
Farnesylated ComX	1362.74	1362.69
Daptomycin	1619.71	1619.37
Farnesylated Daptomycin	1824.32	1824.46

Table **S4.** Summary of observed and calculated MALDI–TOF MS data in this study(**[M+Na]$^+$**).

**Name**	**m/z [M+Na]$^+$** (calculated) (Da)	**m/z [M+Na]$^+$** (observed) (Da)
Daptomycin_Na$^+$	1643.69	1643.19
Farnesylated Daptomycin_Na$^+$	1847.31	1846.44

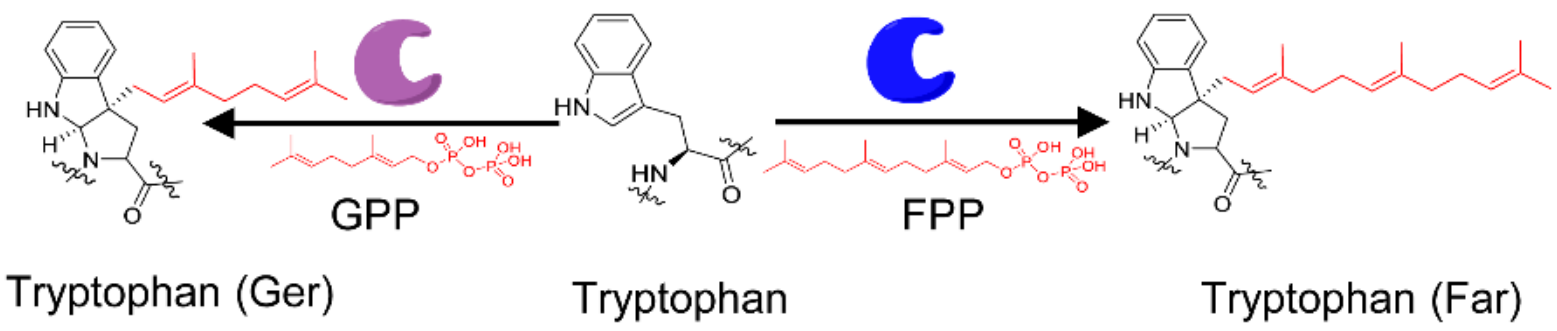

Figure S1. Prenyltransferase-modified Trp residue of ComX pheromones. The structure of the prenyltransferase-modified Trp residue of ComX pheromones. Chemical structures of the Trp residue before (middle), after ComQ-mediated geranylation (left) and after ComQ-mediated farnesylation (right). The blue and purple crescents represent ComQ from different *Bacillus* species. Blue is related to farnesylation, while purple is involved in geranylation.

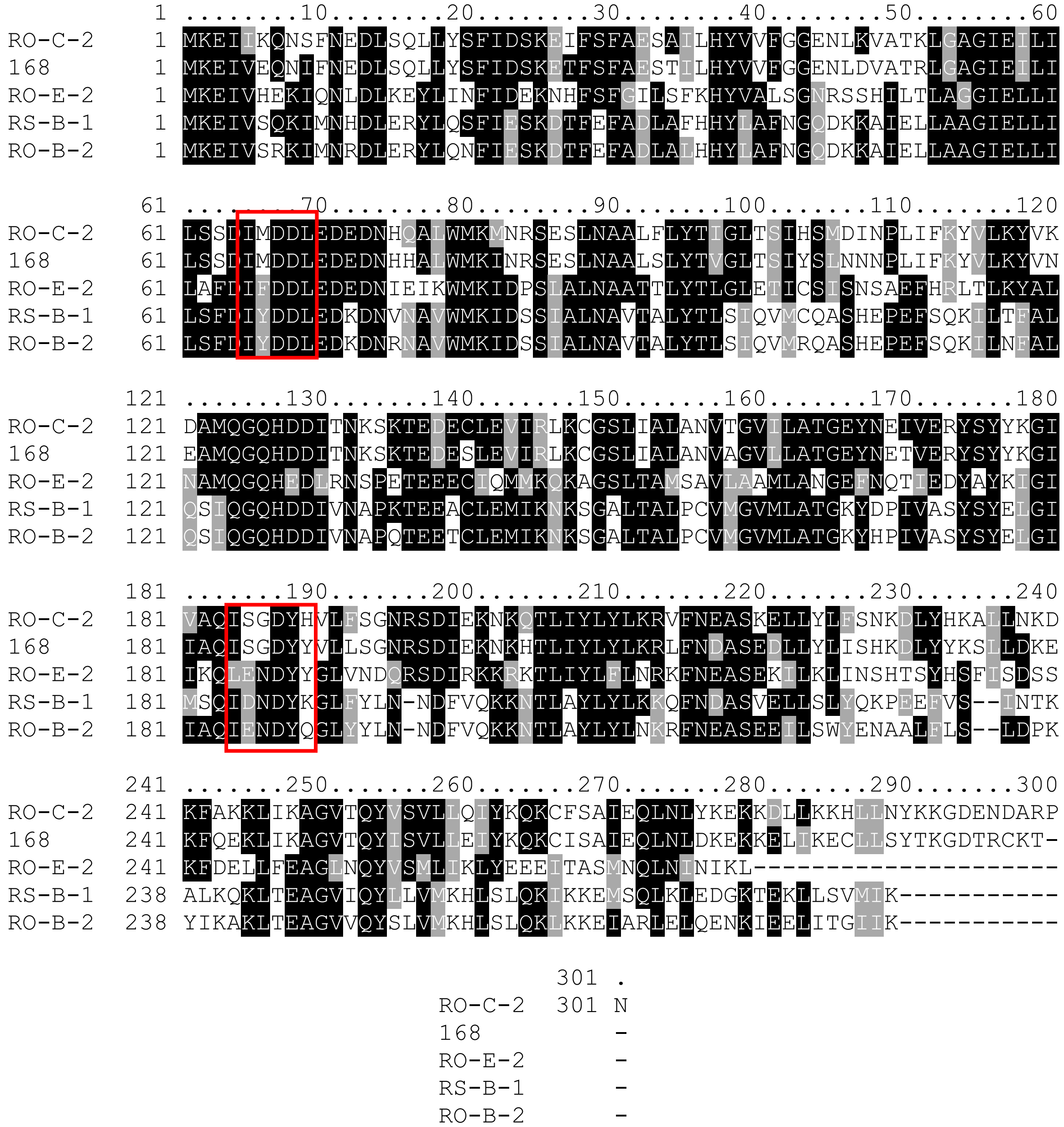

Figure S2. Comparison of amino acid sequences of ComQ from different *Bacillus* species

The accession numbers of the prepursor ComXs from different *bacillus* species are AAL67728 (RO-C-2), AIY94492 (168), AAL67740 (RO-E-2), AAF82173 (RS-B-1), AAL67731 (RO-B-2).

These enzymes contain two conserved aspartate-rich motifs, known as the first aspartate-rich motif (FARM) and the second aspartate-rich motif (SARM), highlighted in the red box of the Figure. The FARM motif in ComQ is highly conserved, whereas the residues corresponding to the SARM motif show considerable variation. Multiple sequence alignment was performed using ClustalW and visualized using ESPript.

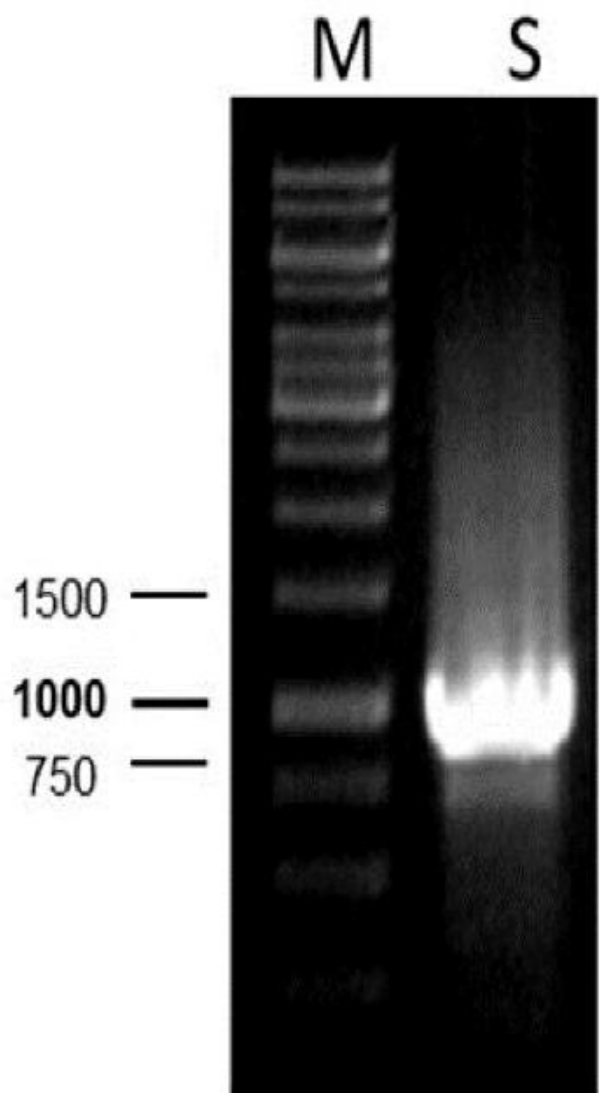

Figure S3. 1.2 % TAE agarose gel electrophoretic analyses for $ComQ_{168}$ amplification. M: 1kb DNA ladder; S: amplified $ComQ_{168}$ sample. The $ComQ_{168}$ gene is 900 bp in length. The nucleotide sequence is provided below.

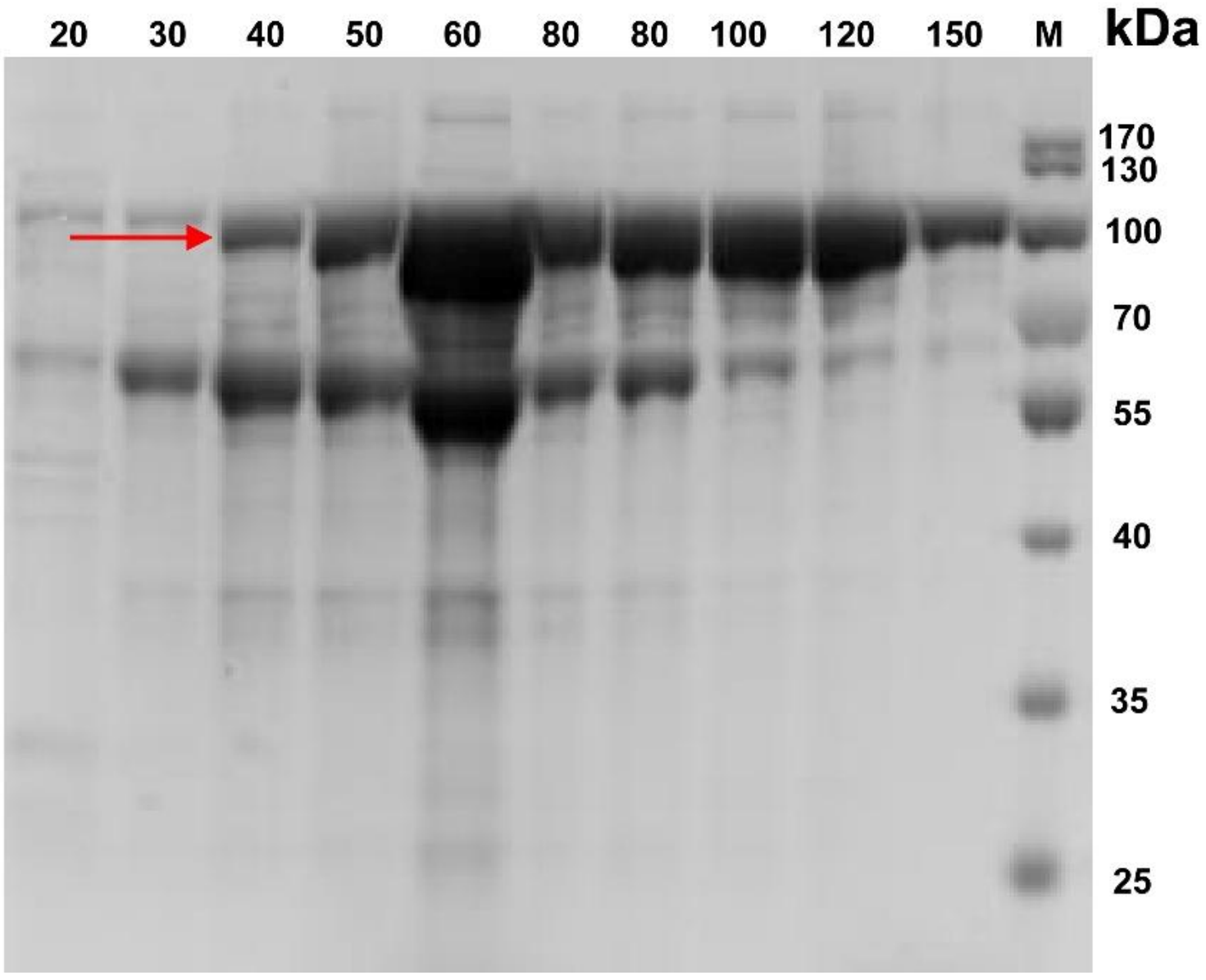

Figure S4. Different concentrations of imidazole elution solution for concentrating pCOLD-TF-$ComQ_{168}$. Numbers from 20 to 150 represent different concentrations of imidazole (mM). $ComQ_{168}$ has an expected molecular weight of ~34 kDa, and the Trigger Factor (TF) chaperone is ~48 kDa. The TF-$ComQ_{168}$ fusion (target) protein is approximately 82 kDa. The red arrow indicates the target protein band.

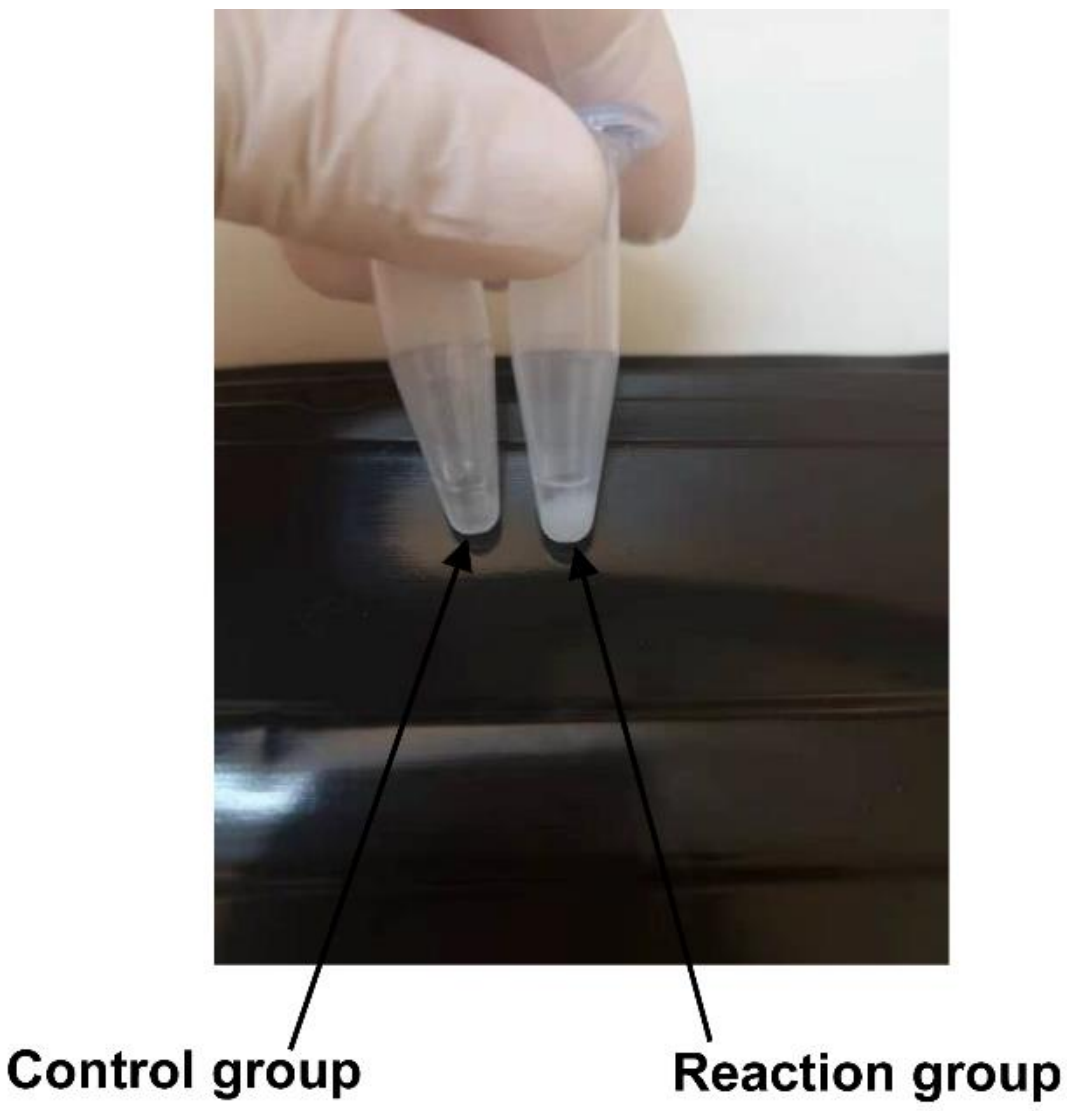

Figure S5. Visual comparison of the *in vitro* farnesylation reaction and the control reaction. After incubation, the reaction mixture containing $ComQ_{168}$ appeared turbid, whereas the control remained relatively clear. The control sample is shown on the left, and the reaction sample on the right. Each reaction had a total volume of 100 µL. The reaction mixture contained 5 mM $MgCl_2$, 5 mM $CaCl_2$, 5 mM daptomycin, 5 mM farnesyl pyrophosphate ammonium salt, and concentrated $ComQ_{168}$ in 20 mM PBS buffer (pH 8.0). In the control reaction, the empty pCOLD-TF plasmid was used in place of $ComQ_{168}$.

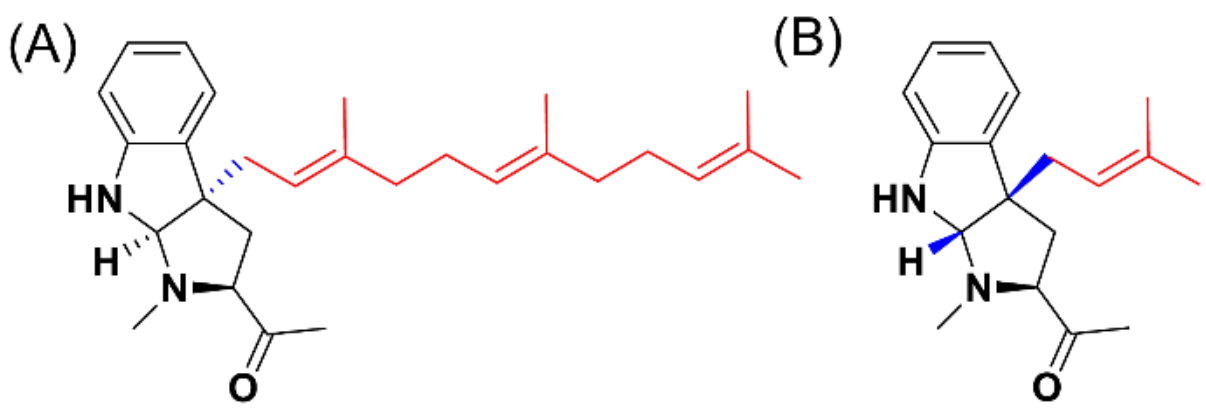

Figure S6. Chemical structure of the modified tryptophan residues by $ComQ_{168}$ and KgpF. Both enzymes generate a similar tricyclic structure as part of their modification, highlighting a shared pattern. However, key differences are also present and are color-coded in the Figure: red indicates differences in the required prenyl donors, while green highlights variations in the stereochemistry structures of the resulting products. (A) modified by $ComQ_{168}$; (B) modified by KgpF.

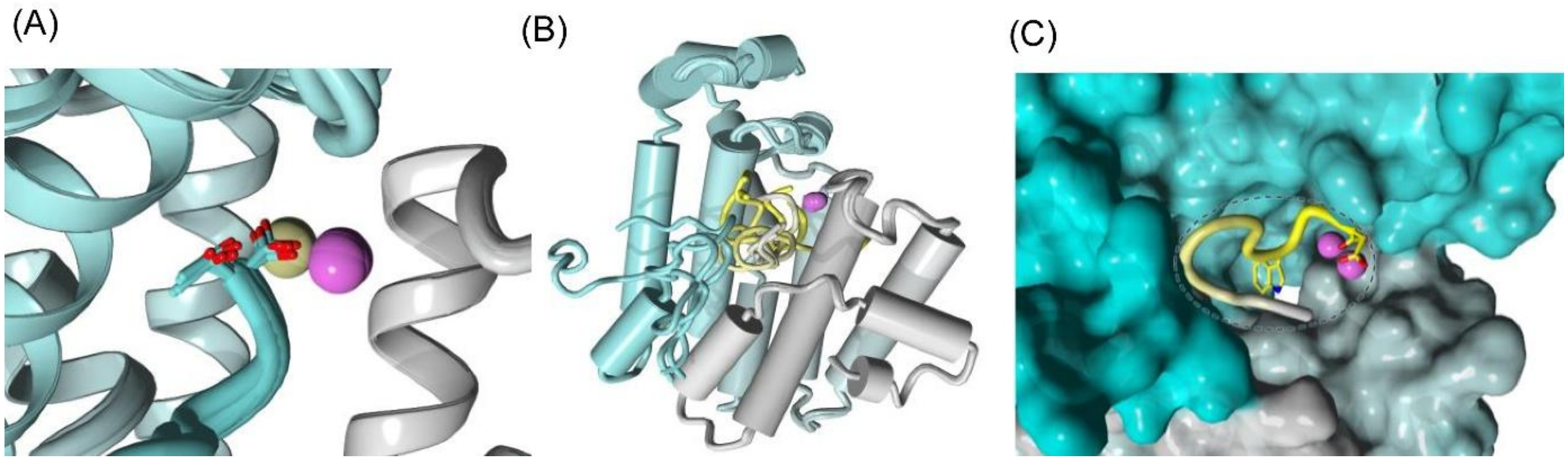

Figure S7. Structural simulation analyses of the $ComQ_{168}$ and its homologous enzymes. Protein structure was predicted using AlphaFold Protein Structure Database. (A) ComQ apo interaction of 2 Mg^{2+} with C terminal carboxy. (B) AF3 models of ComQ in complex with short ComX, in which ComX always displaces ComQ's C-terminus from the active site. (C) AF3 places ComX in the active site in a conformation approximating a circle.

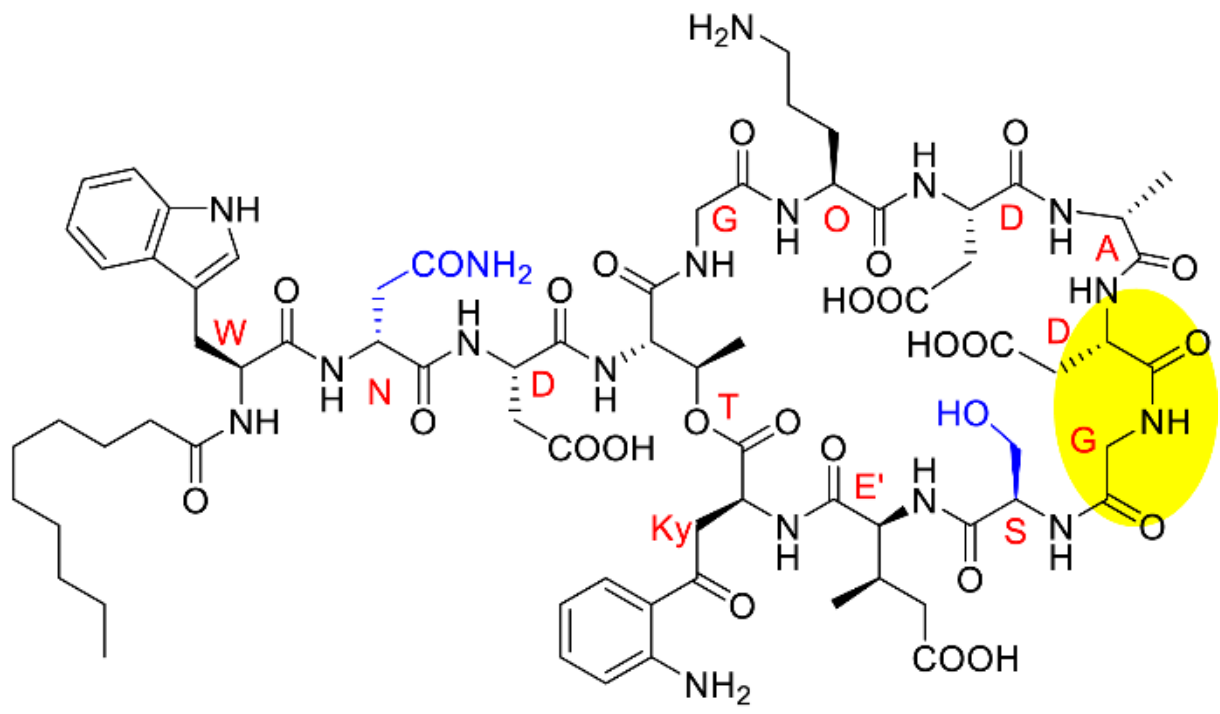

Figure S8. The chemical structure of daptomycin is depicted, with the corresponding amino acids labeled in red. The D amino acid, which were present in blue in the Figure. The DG motif, identical to the C-terminus of ComQ, is highlighted in yellow and the background is also in yellow. Ky: kynurenine, O: ornithine;

> *Bacillus subtilis* 168 | BSU_31710 | *comQ*

ATGAAGGAGATTGTGGAGCAAAACATATTTAACGAGGATTTGTCACAACTTCTTTACTCATTTATTG
ATTCTAAAGAAACATTCTCCTTTGCTGAATCCACAATCTTACATTATGTTGTTTTTGGAGGGGAGAA
TTTAGATGTTGCAACAAGGCTTGGCGCGGGAATAGAAATTCTTATTCTTTCATCAGATATTATGGA
TGATCTTGAAGATGAAGATAATCACCATGCATTATGGATGAAAATTAATCGCTCGGAGTCGTTAAA
TGCAGCACTTTCCTTGTATACAGTAGGATTAACGAGTATTTATTCATTAAATAACAATCCTCTAATT
TTTAAATATGTTTTAAAATACGTAAACGAGGCCATGCAAGGTCAACATGACGATATCACTAATAAAT
CTAAAACAGAAGATGAAAGTTTAGAAGTAATTAGATTAAAATGTGGAAGTCTGATTGCACTAGCAA
ATGTAGCAGGTGTTTTATTGGCTACAGGAGAATATAATGAAACAGTTGAAAGATATTCCTACTATA
AAGGGATTATAGCTCAAATTTCTGGAGATTATTATGTTCTTTTATCCGGAAATCGCAGTGATATTGA
AAAAAACAAACATACACTGATTTACTTATATCTTAAGCGCCTTTTTAATGATGCTTCGGAAGATTTA

TTATACTTAATTTCACATAAAGACTTATATTATAAATCGTTACTTGATAAAGAGAAATTCCAAGAAAA
ATTAATTAAAGCGGGAGTTACTCAATACATTTCGGTATTATTGGAAATATATAAACAGAAATGTATT
TCTGCTATAGAACAATTAAATTTAGATAAAGAAAAAAAAGAGCTAATAAAGGAATGCTTATTAAGTT
ATACAAAGGGGGATACAAGATGCAAGACCTAA

> *Bacillus subtilis* 168 | BSU_31710 | ComQ

MKEIVEQNIFNEDLSQLLYSFIDSKETFSFAESTILHYVVFGGENLDVATRLGAGIEILILSSDIMDDLEDE
DNHHALWMKINRSESLNAALSLYTVGLTSIYSLNNNPLIFKYVLKYVNEAMQGQHDDITNKSKTEDESL
EVIRLKCGSLIALANVAGVLLATGEYNETVERYSYYKGIIAQISGDYYVLLSGNRSDIEKNKHTLIYLYLK
RLFNDASEDLLYLISHKDLYYKSLLDKEKFQEKLIKAGVTQYISVLLEIYKQKCISAIEQLNLDKEKKELIK
ECLLSYTKGDTRCKT
